# Retrospective Efficacy Analysis of Immune Checkpoint Inhibitor Rechallenge in Patients with Non-Small Cell Lung Cancer

**DOI:** 10.3390/jcm9010102

**Published:** 2019-12-31

**Authors:** Yuki Katayama, Takayuki Shimamoto, Tadaaki Yamada, Takayuki Takeda, Takahiro Yamada, Shinsuke Shiotsu, Yusuke Chihara, Osamu Hiranuma, Masahiro Iwasaku, Yoshiko Kaneko, Junji Uchino, Koichi Takayama

**Affiliations:** 1 Department of Pulmonary Medicine, Graduate School of Medical Science, Kyoto Prefectural University of Medicine, Kyoto 602-8566, Japan; ktym2487@koto.kpu-m.ac.jp (Y.K.); m04035ts@koto.kpu-m.ac.jp (T.S.); miwasaku@koto.kpu-m.ac.jp (M.I.); kaneko-y@koto.kpu-m.ac.jp (Y.K.); uchino@koto.kpu-m.ac.jp (J.U.); takayama@koto.kpu-m.ac.jp (K.T.); 2 Department of Pulmonary Medicine, Japanese Red Cross Kyoto Daini Hospital, Kyoto 602-8026, Japan; dyckw344@yahoo.co.jp; 3 Department of Pulmonary Medicine, Matsushita Memorial Hospital, Moriguchi 570-8540, Japan; t-yamada@koto.kpu-m.ac.jp; 4 Department of Pulmonary Medicine, Japanese Red Cross Kyoto Daiichi Hospital, Kyoto 605-0981, Japan; sshiotsu@gmail.com; 5 Department of Pulmonary Medicine, Uji-Tokushukai Medical Center, Uji 611-0041, Japan; c1981311@koto.kpu-m.ac.jp; 6 Department of Pulmonary Medicine, Otsu City Hospital, Otsu 520-0804, Japan; osamu319@true.ocn.ne.jp

**Keywords:** immunotherapy, rechallenge, non-small cell lung cancer, retrospective analysis

## Abstract

Little is known regarding the effectiveness and tolerability of immune checkpoint inhibitor (ICI) rechallenge after disease progression following initial ICI treatments. To identify eligible patients for ICI rechallenge, we retrospectively analyzed the relationship between clinical profiles and the effect of ICI rechallenge in patients with non-small cell lung cancer (NSCLC). We enrolled 35 NSCLC patients at six different institutions who were retreated with ICIs after discontinued initial ICI treatments due to disease progression. Cox proportional hazards models were used to assess the impact of clinical profiles on overall survival (OS) and progression-free survival (PFS). Median PFS and OS were 81 d (95% confidence interval, CI, 41–112 d) and 225 d (95% CI 106–361 d), respectively. The objective response rate was 2.9%, and the disease control rate was 42.9%. Multivariate analysis demonstrated that Eastern Cooperative Oncology Group Performance Score (ECOG-PS) ≥ 2 (hazard ratio, HR, 2.38; 95% CI 1.03–5.52; *p* = 0.043) and body mass index (BMI) > 20 (HR 0.43, 95% CI 0.19–0.95, *p* = 0.036) were significantly associated with PFS of ICI rechallenge. Our observations suggest that poor ECOG-PS and low BMI at intervention with ICI rechallenge may be negative predictors for ICI rechallenge treatment in patients with NSCLC.

## 1. Introduction

Lung cancer is the leading cause of cancer death worldwide [1]. Current clinical studies have shown that some types of molecularly targeted therapies are able to successfully treat a subset of patients with advanced non-small cell lung cancer (NSCLC). In addition, cancer immunotherapies, such as programmed cell death protein 1 (PD-1)/programmed death ligand 1 (PD-L1) checkpoint inhibitors, are being developed as promising alternative strategies for treating patients with advanced NSCLC. Of the current immune checkpoint inhibitors (ICIs), nivolumab, pembrolizumab, atezolizumab, and durvalumab have been approved in the United States, Japan, and other countries for the treatment of patients with NSCLC based on phase III clinical trials [2,3,4,5,6]. However, the majority of patients with NSCLC ultimately acquire resistance to ICI treatments. After acquiring resistance to several therapeutic regimens, ICI rechallenge is considered to be one of the therapeutic options for patients with recurrent NSCLC. Unfortunately, ICI rechallenge treatment has been clinically effective in only a small number of NSCLC patients. Therefore, it is warranted to identify predictive clinical markers for the effectiveness of ICI rechallenge. Previous retrospective studies regarding ICI rechallenge have analyzed only limited numbers of NSCLC patients [7,8]. Hence, little is currently known regarding the effectiveness and tolerability of ICI rechallenge after disease progression following initial ICI treatments. In an effort to identify the patients eligible for ICI rechallenge treatment, we retrospectively analyzed the relationship between the clinical profiles and the effect of ICI rechallenge in patients with NSCLC.

## 2. Experimental Section

### 2.1. Patients

We enrolled 35 patients with NSCLC who were retreated with ICIs after their initial ICI treatments were discontinued due to disease progression. The patients were treated between April 2017 and November 2018 at one of six different institutions, which included University Hospital Kyoto Prefectural University of Medicine (Kyoto, Japan), Japanese Red Cross Kyoto Daiichi Hospital (Kyoto, Japan), Japanese Red Cross Kyoto Daini Hospital (Kyoto, Japan), Uji-Tokushukai Medical Center (Kyoto, Japan), Matsushita Memorial Hospital (Osaka, Japan), and Otsu City Hospital (Shiga, Japan). Patient clinical data were retrospectively obtained from their medical records, including age, sex, height, weight, body mass index (BMI) at the start of ICI rechallenge, histological subtype, PD-L1 expression level in tumors, epidermal growth factor receptor (EGFR) mutation status, disease staging, metastatic site, corticosteroid administration, Eastern Cooperative Oncology Group Performance Status (ECOG-PS), smoking status, laboratory findings at the time of ICI rechallenge, and overall survival (OS), progression-free survival (PFS), response rate, and disease control rate for the patients receiving ICI treatment based on the Response Evaluation Criteria in Solid Tumors (RECIST; version 1.1). The study protocol was approved by the ethics committee of each hospital. Tumor–node–metastasis (TNM) stage was classified using the TNM stage classification system, version 8. Six received 2.5 mg to 10 mg p.o. of corticosteroids administration due to improvement in the cachexia. We have added this information in the materials and methods section. This study is an exploratory trial.

### 2.2. Tumor PD-L1 Analysis

PD-L1 expression was analyzed by SRL, Inc. using a PD-L1 IHC 22C3 pharmDx assay (Agilent Technologies, Santa Clara, CA, USA). The PD-L1 tumor proportion score (TPS) was calculated as a percentage of at least 100 viable tumor cells with complete or partial membrane staining. Pathologists at SRL, Inc. interpreted the TPS results.

### 2.3. Statistical Analysis

Statistical analyses were performed using EZR statistical software, version 1.30 [9]. All statistical tests were two-sided, and *p*-values < 0.05 were regarded as statistically significant. The cutoff values for body mass index (BMI), albumin, lactate dehydrogenase (LDH), neutrophil to lymphocyte ratio (NLR), lymphocyte to monocyte ratio (LMR), platelet to lymphocyte ratio (PLR), and C-reactive protein (CRP) following prior therapy were determined according to previous reports [10,11,12,13,14,15]. The PFS and OS were calculated using the Kaplan–Meier method, and differences were compared using the log-rank test. The hazard ratios (HRs) and their 95% confidence intervals (CIs) were estimated using the Cox proportional hazards model in univariate analyses. The Cox proportional hazards model was also used for the multivariate analyses.

## 3. Results

### 3.1. Patient Characteristics

A total of 35 NSCLC patients treated with ICI rechallenge between April 2017 and November 2018 at six different institutions in Japan were enrolled. The median age was 70 years (range: 40–83 years), 24 patients (68.6%) were male, and 27 (77.1%) patients had a history of smoking. The histological subtypes were (23, i.e., 65.7%) adenocarcinoma and (10, i.e., 28.6%) squamous cell carcinoma. Metastatic disease was detected in the liver of five patients (14.3%) and in the brain of seven patients (20%). Of the patients, 10 (28.6%) had stage III disease, 19 (54.3%) had stage IV disease, and six (17.1%) had postoperative recurrence at the time of intervention with the initial ICI treatment. An EGFR mutation was detected in four patients (11.4%). There were no ALK-positive patients. ECOG-PS was 0–1 for 23 patients (65.7%) and 2–4 for 12 patients (34.3%). The PD-L1 TPS was ≥50% for 14 patients (50%), 1–49% for eight patients (7%), <0% for seven patients (29%), and not evaluated for six patients (17.1%). The BMI was ≥25 for 3 patients (8.6%), 20–25% for 16 patients (45.7%), and <20 for 16 patients (45.7%). Table 1 shows the baseline characteristics of patients. 

### 3.2. Efficacy and Safety of ICI Treatments

**Figure 1:**
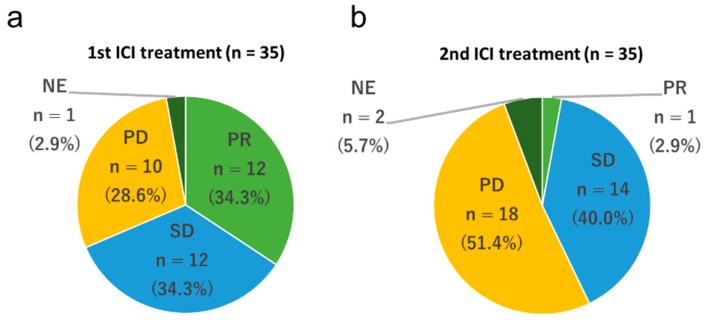
Frequency of the best overall response to immune checkpoint inhibitors (ICIs). (**a**) Frequency of the best overall response to first ICI treatment. (**b**) Frequency of the best overall response to ICI rechallenge treatment. PD, progressive disease; PR, partial response; SD, stable disease; NE, not evaluated.

**Figure 2:**
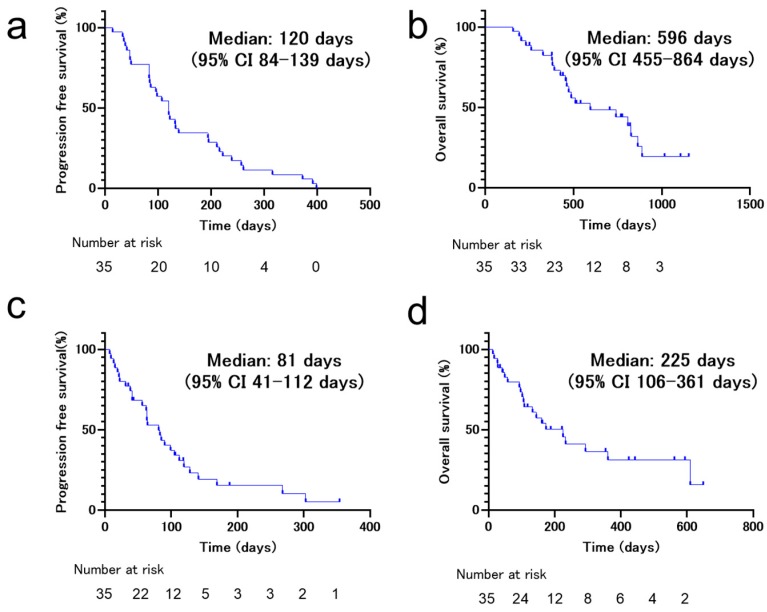
Kaplan–Meier survival curves of progression-free survival (PFS) and overall survival (OS) of patients who received immune checkpoint inhibitor (ICI) rechallenge treatment. (**a**) PFS of non-small cell lung cancer (NSCLC) patients (*n* = 35) on first ICI treatment. (**b**) OS of NSCLC patients (*n* = 35) on first ICI treatment. (**c**) PFS of NSCLC patients (*n* = 35) on ICI rechallenge treatment. (**d**) OS of NSCLC patients (*n* = 35) on ICI rechallenge treatment.

The initial ICI treatment consisted of nivolumab for 19 (54.3%) patients, pembrolizumab for 12 (34.3%) patients, and atezolizumab for four (11.4%) patients. The rechallenge treatment consisted of nivolumab for five (14.3%) patients, pembrolizumab for 7 (20.0%) patients, and atezolizumab for 23 (65.7%) patients. The patients were treated with different regimens of ICIs between the initial and rechallenge treatments. In the initial ICI treatment, no patients experienced a complete response (0%), 12 experienced a partial response (34.3%), 12 experienced stable disease (34.3%), 10 experienced progressive disease (28.6%), and one was non-evaluable (2.9%). The objective response rate was 34.3%, and the disease control rate was 68.6% (Figure 1a). The PFS and OS of the initial ICI treatments were 120 d (95% CI 84–139 d) and 596 d (95% CI 455–864 d), respectively (Figure 2a,b). In the ICI rechallenge treatment, no patients experienced a complete response (0%), one experienced a partial response (2.9%), 14 experienced stable disease (40.0%), 18 experienced progressive disease (51.4%), and two were non-evaluable (5.7%). The objective response rate was 2.9% and the disease control rate was 45.7% (Figure 1b). The PFS and OS of the ICI rechallenge were 81 d (95% CI 41–112 d) and 225 d (95% CI 106–361 d), respectively (Figure 2c,d). 

**Figure 3:**
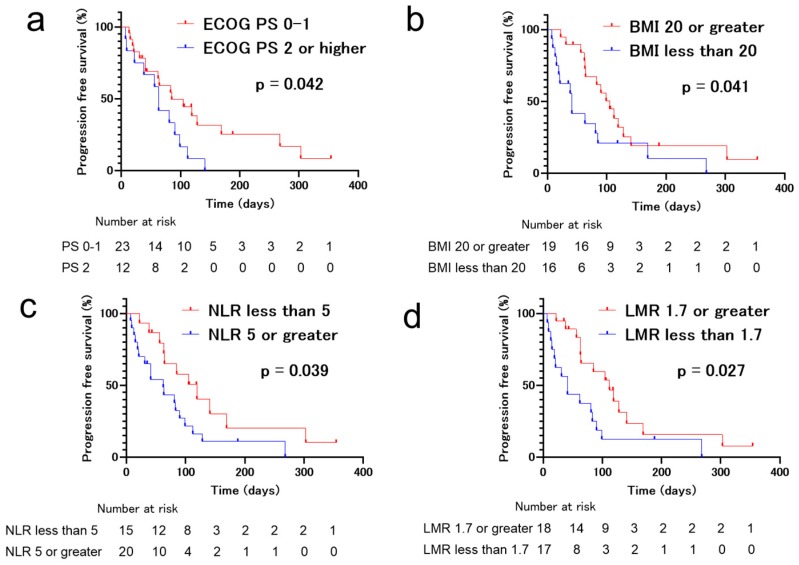
Kaplan–Meier survival curves for progression-free survival (PFS) of patients who received immune checkpoint inhibitor (ICI) rechallenge treatment. (**a**) Eastern Cooperative Oncology Group (ECOG-PS) ≥ 2, (**b**) body mass index (BMI) ≤ 20, (**c**) neutrophil-to-lymphocyte ratio (NLR) > 5, and (**d**) lymphocyte-to-monocyte ratio (LMR) ≤ 1.7 were significantly associated with inferior PFS.

Univariate analyses of the patient data revealed that ECOG-PS ≥ 2 (HR 2.21, 95% CI 1.00–4.83, *p* = 0.048), BMI > 20 (HR 0.47, 95% CI 0.22–0.99, *p* = 0.047), NLR ≥ 5 (HR 2.22, 95% CI 1.02–4.84, *p* = 0.045), and LMR < 1.7 (HR 0.44, 95% CI 0.21–0.93, *p* = 0.032) were significantly associated with PFS of ICI rechallenge (Table 2). Moreover, multivariate analysis demonstrated that ECOG-PS ≥ 2 (HR 2.38, 95% CI 1.03–5.52, *p* = 0.043) and BMI > 20 (HR 0.43, 95% CI 0.19–0.95, *p* = 0.036) were significantly associated with PFS of ICI rechallenge (Table 3 and Figure 3). 

Univariate analyses of the patient data revealed that ECOG-PS ≥ 2 (HR 4.23, 95% CI 1.65–10.89, *p* = 0.0023), CRP > 1.0 (HR 2.92, 95% CI 1.10–7.76, *p* = 0.032), albumin > 3.5 (HR 0.37, 95% CI 0.15–0.90, *p* = 0.028), and PLR > 262 (HR 2.80, 95% CI 1.02–7.67, *p* = 0.045) were significantly associated with OS of ICI rechallenge (Table 2). Multivariate analysis demonstrated that ECOG-PS ≥ 2 (HR 3.01, 95% CI 1.10–8.24, *p* = 0.032) was significantly associated with OS of ICI rechallenge (Table 3).

## 4. Discussion

PD-L1 expression in tumors has been used clinically as a positive predictive biomarker for the effective initial ICI treatment of patients with NSCLC [16]. However, clinically useful biomarkers have not yet been identified for predicting the efficacy of ICI rechallenge. Fujita et al. reported that objective response rate (ORR), disease control rate (DCR), and PFS values of pembrolizumab rechallenge after refractory nivolumab for 12 patients with NSCLC were 8.3%, 41.7%, and 3.1 months, respectively [8]. In addition, ORR, DCR, and PFS of atezolizumab rechallenge after refractory anti-PD-1 antibodies for 18 patients with NSCLC were 0%, 38.9%, and 2.9 months, respectively [17]. Another report showed that ORR, DCR, and PFS values of ICI rechallenge in 14 patients with ICI refractory tumors were 7.1%, 21.4%, and 1.6 months, respectively [7]. Our current observations showed that ORR, DCR, PFS, and OS values of ICI rechallenge in 35 patients with NSCLC were 2.9%, 42.9%, 2.7 months, and 7.5 months, respectively. These reproducible findings suggest that refractory NSCLC tumors for initial ICI treatments may exhibit poor responses to ICI rechallenge treatments, and the clinical benefits may be limited compared with those of the initial ICI treatment. However, a subset of patients with NSCLC demonstrate good outcomes with ICI rechallenge treatments. Therefore, there is a need for the elucidation of predictive clinical factors for re-treatment of ICI responders among patients with NSCLC. 

Our multivariate analysis identified ECOG-PS and BMI as independent factors associated with poorer PFS of ICI rechallenge treatment in patients with NSCLC who were refractory to initial ICI treatment. This is the first report that identifies predictive clinical factors for the efficacy of ICI rechallenge in patients with NSCLC. The general and nutritional status of patients with NSCLC are closely related to the effects of ICI treatment. Several studies have demonstrated that poor ECOG-PS is a predictive negative factor related to clinical outcomes of initial ICI treatment in patients with NSCLC [10,13,18,19]. ECOG-PS is one of the factors that determines the tumor immune environment, and it has been reported that an imbalance of circulating T-lymphocyte subpopulations in patients with gastric cancer correlates with ECOG-PS [20]. BMI is widely used for relating weight to height, defining body size, and indicating nutritional status. In addition, a lower BMI is associated with increased mortality risk [21,22,23,24]. Our previous clinical study demonstrated that NSCLC patients with sarcopenia exhibit a significantly shorter median PFS following ICI treatment compared to that of non-sarcopenia patients [25]. Given these observations, a poor ECOG-PS and a low BMI at the time of ICI treatment intervention may be useful for predicting non-responders to initial ICI treatment, as well as ICI rechallenge treatment among patients with NSCLC. Recent clinical trials demonstrated that the ghrelin/growth hormone secretagogue receptor agonist anamorelin increases lean body mass and improves the performance status in NSCLC patients with cachexia [26,27]. Therefore, the administration of anamorelin may improve the effect of ICI rechallenge treatment.

The effectiveness of initial ICI treatments has been reported to be associated with PD-L1 expression in NSCLC tumors [28]. Our current observations show that the patients with PD-L1 expression tended to have longer PFS. This suggests that PD-L1 expression levels in pre-treatment tumors may be a factor to consider when with regard to ICI rechallenge treatment. Regardless, based on our observations, the values of blood NLR, LMR, and PLR at baseline may be useful tools for predicting responders to the ICI rechallenge treatment, which is consistent with the initial ICI treatment [10,13,15,29]. Thus, when considering ICI rechallenge treatment in patients with NSCLC, the inflammation markers, such as NLR, LMR, and PLR, may be useful to some extent for identifying responders to ICI rechallenge.

A previous report suggests that the response to initial ICI treatments correlates with the clinical response to ICI rechallenge treatment in patients with melanoma [30]. However, our results failed to indicate a relationship between clinical outcomes of initial ICI treatment and ICI rechallenge treatment in patients with NSCLC. This suggested that there may be differences between the immunological properties of NSCLC and melanoma.

The current study had several limitations. First, it consisted of a small retrospective sample. Therefore, a further large-cohort study is warranted to identify the predictive markers of ICI rechallenge treatment. Second, although the treatment was administered at multiple centers, there may have been bias in terms of the timing of evaluating the patients using CT scanning, even though it was performed every 1–3 months after treatment.

## 5. Conclusions

Our observations suggest that a poor ECOG-PS and a low BMI at the time of intervention with ICI rechallenge may be useful as negative predictors for ICI rechallenge treatment in patients with NSCLC. As this retrospective study was a relatively small-scale study, further experiments are needed to validate the observations.

## Figures and Tables

**Table 1:** Patient characteristics at immune checkpoint inhibitor (ICI) rechallenge treatment.

Items	Group	*n* (%)
Age	Median (range)	70 (48–83)
Gender	Male	24 (68.6)
	Female	11 (31.4)
Eastern Cooperative Oncology Group Performance Score (ECOG-PS)	0–1	23 (65.7)
	2–4	12 (34.3)
Histology	Adenocarcinoma	23 (65.7)
	Squamous cell carcinoma	10 (28.6)
	Other	2 (5.7)
Smoking Status	Never smoker	8 (22.9)
	Current or former smoker	27 (77.1)
Staging	Stage III	10 (28.6)
	Stage IV	19 (54.3)
	Postoperative recurrence	6 (17.1)
Epidermal Growth Factor Receptor (EGFR) Mutations	Positive	4 (11.4)
	Negative	31 (88.6)
PD-L1 tumor proportion score (TPS)	≥50%	14 (40)
	1–49%	8 (22.9)
	<1%	7 (20)
	Not evaluated	6 (17.1)
Metastasis	Liver metastasis	5 (14.3)
	Brain metastasis	7 (20)
Body Mass Index (BMI)	BMI > 25	3 (8.6)
	25 ≥ BMI > 20	16 (45.8)
	BMI ≤ 20	16 (45.8)
Corticosteroid Administration	Yes	6 (17.1)
	No	29 (82.9)
History of Treatment before ICI Rechallenge	Surgery	6 (17.1)
	Radiation therapy	12 (34.3)
	Chemotherapy (platinum)	30 (85.7)
	Chemotherapy (non-platinum)	25 (71.4)
First ICIs	Nivolumab	19 (54.3)
	Pembrolizumab	12 (34.3)
	Atezolizumab	4 (11.4)
Second ICIs	Nivolumab	5 (14.3)
	Pembrolizumab	7 (20)
	Atezolizumab	23 (65.7)
Line of First ICI	Median (range)	3 (1–15)
Line of Second ICI	Median (range)	4 (2–19)
Duration from the End of the First ICI to the Start of the Second ICI	Median (95% confidence interval; CI)	157 d (106–238)

**Table 2:** Cox proportional hazards and logistic regression models for progression-free survival (PFS) and overall survival (OS).

Items	PFS (Univariate Analysis)		OS (Univariate Analysis)	
	HR (95% CI)	*p*-Value	HR (95% CI)	*p*-Value
Age > 75 Years	0.81 (0.34–1.91)	0.63	1.45 (0.55–3.80)	0.45
Male Gender	1.47 (0.66–3.26)	0.35	2.39 (0.80–7.17)	0.12
Smoker	1.508 (0.63–3.59)	0.35	2.50 (0.73–8.58)	0.14
ECOG-PS ≥ 2	2.21 (1.00–4.83)	0.048	4.23 (1.65–10.89)	0.0028
Squamous Histology	1.08 (0.47–2.48)	0.86	0.67 (0.24–1.83)	0.43
EGFR Mutations Positive	0.83 (0.28–2.43)	0.73	1.17 (0.34–4.02)	0.80
BMI > 20	0.47 (0.22–0.99)	0.047	0.42 (0.17–1.02)	0.056
BMI > 25	0.54 (0.19–1.59)	0.27	0.92 (0.26–3.25)	0.90
Corticosteroids Administration	1.3 (0.49–3.52)	0.58	0.66 (0.19–2.27)	0.51
Alb > 3.5 g/dL	0.53 (0.25–1.11)	0.092	0.37 (0.15–0.90)	0.028
CRP > 1.0 mg/dL	1.44 (0.68–3.04)	0.34	2.92 (1.10–7.76)	0.032
LDH > 245 U/L	1.41 (0.67–2.99)	0.37	2.16 (0.89–5.24)	0.090
NLR > 5.0	2.22 (1.02–4.84)	0.045	1.98 (0.79–4.92)	0.14
LMR > 1.7	0.44 (0.21–0.93)	0.032	0.51 (0.21–1.23)	0.14
PLR > 262	2.23 (0.99–5.03)	0.054	2.80 (1.02–7.67)	0.045
Liver Metastasis	1.79 (0.61–5.28)	0.29	1.95 (0.55–6.886)	0.30
Brain Metastasis	1.17 (0.47–2.91)	0.73	0.58 (0.17–2.00)	0.39
PD-L1 TPS 1–49%	0.32 (0.096–1.05)	0.059	0.55 (0.16–1.89)	0.34
PD-L1 TPS > 50%	0.35 (0.12–1.05)	0.061	0.42 (0.12–1.49)	0.18
Lines between First and Second ICIs > 2	1.26 (0.55–2.87)	0.58	1.54 (0.59–4.04)	0.38
PFS of First ICI >120 d	1.06 (0.50–2.23)	0.89	1.30 (0.55–3.08)	0.54
Duration from the End of the First ICI to the Second ICI >157 d	0.97 (0.47–2.02)	0.94	0.77 (0.32–1.84)	0.55
Partial Response with First ICIs	0.58 (0.26–1.33)	0.20	0.99 (0.40–2.48)	0.98

**Table 3:** Cox proportional hazards and logistic regression models for progression-free survival (PFS) and overall survival (OS).

Items	PFS (Multivariate Analysis)		OS (Multivariate Analysis)	
	HR (95% CI)	*p*-Value	HR (95% CI)	*p*-Value
ECOG-PS ≥ 2	2.38(1.03–5.52)	0.043	3.01(1.10–8.24)	0.032
BMI > 20	0.43(0.19–0.95)	0.036		
Alb > 3.5 g/dL			0.48(0.18–1.28)	0.14
CRP > 1.0 mg/dL			1.51(0.48–4.75)	0.49
NLR > 5.0	1.08(0.22–5.18)	0.93		
LMR > 1.7	0.57(0.13–2.54)	0.46		
PLR > 262			1.93(0.68–5.43)	0.22

## Abbreviations

NSCLC, non-small cell lung cancer; PD-1, programmed cell death protein 1; PD-L1, programmed death-ligand 1; ICIs, immune checkpoint inhibitors; PFS, progression-free survival; OS, overall survival; BMI, body mass index; CRP, C-reaction protein; NLR, neutrophil to lymphocyte ratio; LMR, lymphocyte to monocyte ratio; PLR, platelet to lymphocyte ratio; RECIST, Response Evaluation Criteria in Solid Tumors; CR, complete response; PR, partial response; SD, stable disease; PD, progressive disease; NE, not evaluate; PS, Eastern Cooperative Oncology Group Performance Status; CI, confidence interval; HR, hazard ratio; TPS, tumor proportion score; EGFR, epidermal growth factor receptor.

## References

[1] Miller K.D., Goding Sauer A., Ortiz A.P., Fedewa S.A., Pinheiro P.S., Tortolero-Luna G., Martinez-Tyson D., Jemal A., Siegel R.L. (2018). Cancer Statistics for Hispanics/Latinos, 2018. CA Cancer J. Clin..

[2] Borghaei H., Paz-Ares L., Horn L., Spigel D.R., Steins M., Ready N.E., Chow L.Q., Vokes E.E., Felip E., Holgado E. (2015). Nivolumab versus Docetaxel in Advanced Nonsquamous Non-Small-Cell Lung Cancer. N. Engl. J. Med..

[3] Brahmer J., Reckamp K.L., Baas P., Crino L., Eberhardt W.E., Poddubskaya E., Antonia S., Pluzanski A., Vokes E.E., Holgado E. (2015). Nivolumab versus Docetaxel in Advanced Squamous-Cell Non-Small-Cell Lung Cancer. N. Engl. J. Med..

[4] Herbst R.S., Baas P., Kim D.W., Felip E., Perez-Gracia J.L., Han J.Y., Molina J., Kim J.-H., Arvis C.D., Ahn M.-J. (2016). Pembrolizumab versus docetaxel for previously treated, PD-L1-positive, advanced non-small-cell lung cancer (KEYNOTE-010): A Randomised Controlled Trial. Lancet.

[5] Rittmeyer A., Barlesi F., Waterkamp D., Park K., Ciardiello F., von Pawel J., Gadgeel S.M., Hida T., Kowalski D.M., Dols M.C. (2017). Atezolizumab versus docetaxel in patients with previously treated non-small-cell lung cancer (OAK): A phase 3, open-label, multicentre randomised controlled trial. Lancet.

[6] Antonia S.J., Villegas A., Daniel D., Vicente D., Murakami S., Hui R., Yokoi T., Chiappori A., Lee K.H., Wit de M. (2017). Durvalumab after Chemoradiotherapy in Stage III Non-Small-Cell Lung Cancer. N. Engl. J. Med..

[7] Watanabe H., Kubo T., Ninomiya K., Kudo K., Minami D., Murakami E., Ochi N., Ninomiya T., Harada D., Yasugi M. (2019). The effect and safety of immune checkpoint inhibitor rechallenge in non-small cell lung cancer. Jpn. J. Clin. Oncol..

[8] Fujita K., Uchida N., Kanai O., Okamura M., Nakatani K., Mio T. (2018). Retreatment with pembrolizumab in advanced non-small cell lung cancer patients previously treated with nivolumab: Emerging reports of 12 cases. Cancer Chemother. Pharmacol..

[9] Kanda Y. (2013). Investigation of the freely available easy-to-use software ’EZR’ for medical statistics. Bone Marrow Transpl..

[10] Bagley S.J., Kothari S., Aggarwal C., Bauml J.M., Alley E.W., Evans T.L., Kosteva J.A., Ciunci C.A., Gabriel P.E., Thompson J.C. (2017). Pretreatment neutrophil-to-lymphocyte ratio as a marker of outcomes in nivolumab-treated patients with advanced non-small-cell lung cancer. Lung Cancer.

[11] Kondo T., Nomura M., Otsuka A., Nonomura Y., Kaku Y., Matsumoto S., Muto M. (2019). Predicting marker for early progression in unresectable melanoma treated with nivolumab. Int. J. Clin. Oncol..

[12] Inomata M., Hirai T., Seto Z., Tokui K., Taka C., Okazawa S., Kambara K., Ichikawa T., Imanishi S., Yamada T. (2018). Clinical Parameters for Predicting the Survival in Patients with Squamous and Non-squamous-cell NSCLC Receiving PD-1 Inhibitor Therapy. Pathol. Oncol. Res..

[13] Diem S., Schmid S., Krapf M., Flatz L., Born D., Jochum W., Templeton A.J., Fruh M. (2017). Neutrophil-to-Lymphocyte ratio (NLR) and Platelet-to-Lymphocyte ratio (PLR) as prognostic markers in patients with non-small cell lung cancer (NSCLC) treated with nivolumab. Lung Cancer.

[14] Liu J., Li S., Zhang S., Liu Y., Ma L., Zhu J., Xin Y., Wang Y., Yang C., Cheng Y. (2019). Systemic immune-inflammation index, neutrophil-to-lymphocyte ratio, platelet-to-lymphocyte ratio can predict clinical outcomes in patients with metastatic non-small-cell lung cancer treated with nivolumab. J. Clin. Lab. Anal..

[15] Failing J.J., Yan Y., Porrata L.F., Markovic S.N. (2017). Lymphocyte-to-monocyte ratio is associated with survival in pembrolizumab-treated metastatic melanoma patients. Melanoma Res..

[16] Reck M., Rodriguez-Abreu D., Robinson A.G., Hui R., Csoszi T., Fulop A., Gottfried M., Peled N., Tafreshi A., Cuffe S. (2016). Pembrolizumab versus Chemotherapy for PD-L1-Positive Non-Small-Cell Lung Cancer. N. Engl. J. Med..

[17] Fujita K., Uchida N., Yamamoto Y., Kanai O., Okamura M., Nakatani K., Sawai S., Mio T. (2019). Retreatment With Anti-PD-L1 Antibody in Advanced Non-small Cell Lung Cancer Previously Treated With Anti-PD-1 Antibodies. Anticancer Res..

[18] Mezquita L., Auclin E., Ferrara R., Charrier M., Remon J., Planchard D., Ponce S., Ares L.P., Leroy L., Audigier-Valette C. (2018). Association of the Lung Immune Prognostic Index With Immune Checkpoint Inhibitor Outcomes in Patients With Advanced Non-Small Cell Lung Cancer. JAMA Oncol..

[19] Katayama Y., Yamada T., Tanimura K., Yoshimura A., Takeda T., Chihara Y., Tamiya N., Kaneko Y., Uchino J., Takayama K. (2019). Impact of bowel movement condition on immune checkpoint inhibitor efficacy in patients with advanced non-small cell lung cancer. Thorac. Cancer.

[20] Wang L., Shen Y. (2013). Imbalance of circulating T-lymphocyte subpopulation in gastric cancer patients correlated with performance status. Clin. Lab..

[21] Naik G.S., Waikar S.S., Johnson A.E.W., Buchbinder E.I., Haq R., Hodi F.S., Schoenfeld J.D., Ott P.A. (2019). Complex inter-relationship of body mass index, gender and serum creatinine on survival: Exploring the obesity paradox in melanoma patients treated with checkpoint inhibition. J. Immunother. Cancer.

[22] Cortellini A., Bersanelli M., Buti S., Cannita K., Santini D., Perrone F., Giusti R., Tiseo M., Michiara M., Marino P.D. (2019). A multicenter study of body mass index in cancer patients treated with anti-PD-1/PD-L1 immune checkpoint inhibitors: When overweight becomes favorable. J. Immunother. Cancer.

[23] Xu H., Cao D., He A., Ge W. (2019). The prognostic role of obesity is independent of sex in cancer patients treated with immune checkpoint inhibitors: A pooled analysis of 4090 cancer patients. Int. Immunopharmacol..

[24] De Giorgi U., Procopio G., Giannarelli D., Sabbatini R., Bearz A., Buti S., Basso U., Mitterer M., Ortega C., Bidoli P. (2019). Association of Systemic Inflammation Index and Body Mass Index with Survival in Patients with Renal Cell Cancer Treated with Nivolumab. Clin. Cancer Res..

[25] Nishioka N., Uchino J., Hirai S., Katayama Y., Yoshimura A., Okura N., Tanimura K., Hirai S., Imabayashi T., Chihara Y. (2019). Association of Sarcopenia with and Efficacy of Anti-PD-1/PD-L1 Therapy in Non-Small-Cell Lung Cancer. J. Clin. Med..

[26] Katakami N., Uchino J., Yokoyama T., Naito T., Kondo M., Yamada K., Kitajima H., Yoshimori K., Sato K., Saito H. (2018). Anamorelin (ONO-7643) for the treatment of patients with non-small cell lung cancer and cachexia: Results from a randomized, double-blind, placebo-controlled, multicenter study of Japanese patients (ONO-7643-04). Cancer.

[27] Takayama K., Katakami N., Yokoyama T., Atagi S., Yoshimori K., Kagamu H., Saito H., Takiguchi Y., Aoe K., Koyama A. (2016). Anamorelin (ONO-7643) in Japanese patients with non-small cell lung cancer and cachexia: Results of a randomized phase 2 trial. Support Care Cancer.

[28] Grizzi G., Caccese M., Gkountakos A., Carbognin L., Tortora G., Bria E., Pilotto S. (2017). Putative predictors of efficacy for immune checkpoint inhibitors in non-small-cell lung cancer: Facing the complexity of the immune system. Expert Rev. Mol. Diagn..

[29] Jiang T., Qiao M., Zhao C., Li X., Gao G., Su C., Ren S., Zhou C. (2018). Pretreatment neutrophil-to-lymphocyte ratio is associated with outcome of advanced-stage cancer patients treated with immunotherapy: A meta-analysis. Cancer Immunol. Immunother..

[30] Nomura M., Otsuka A., Kondo T., Nagai H., Nonomura Y., Kaku Y., Matsumoto S., Muto M. (2017). Efficacy and safety of retreatment with nivolumab in metastatic melanoma patients previously treated with nivolumab. Cancer Chemother. Pharmacol..

